# Advancements in Modern Treatment Approaches for Central Post-Stroke Pain: A Narrative Review

**DOI:** 10.3390/jcm13185377

**Published:** 2024-09-11

**Authors:** Auste Asadauskas, Andrea Stieger, Markus M. Luedi, Lukas Andereggen

**Affiliations:** 1 Department of Neurosurgery, Cantonal Hospital of Aarau, 5001 Aarau, Switzerland; 2 Faculty of Medicine, University of Bern, 3012 Bern, Switzerland; 3 Department of Anaesthesiology, Rescue- and Pain Medicine, Cantonal Hospital of St. Gallen, 9007 St. Gallen, Switzerland; 4 Department of Anaesthesiology and Pain Medicine, Inselspital, Bern University Hospital, University of Bern, 3010 Bern, Switzerland

**Keywords:** central post-stroke pain, stroke, medical treatment, intervention, surgery

## Abstract

Purpose of Review: Central post-stroke pain (CPSP) poses a multifaceted challenge in medical practice, necessitating a thorough and multidisciplinary approach for its diagnosis and treatment. This review examines current methods for addressing CPSP, highlighting both pharmacological and non-pharmacological therapies. It covers the mechanisms and clinical effectiveness of these treatments in managing CPSP and emphasizes the importance of personalized treatment plans, given the varied causes of CPSP. Recent Findings: Recent advancements have illuminated diverse treatment modalities for CPSP. Pharmacotherapy spans from conventional analgesics to anticonvulsants and antidepressants, tailored to mitigate the neuropathic characteristics of CPSP. Non-pharmacological interventions, including physical therapy and psychological strategies, are pivotal in managing CPSP’s chronic nature. For cases resistant to standard treatments, advanced interventions such as nerve blocks and surgical procedures like deep brain stimulation (DBS) or motor cortex stimulation (MCS) are considered. Additionally, innovative technologies such as neuromodulation techniques and personalized medicine are emerging as promising avenues to enhance therapeutic outcomes and improve quality of life for individuals grappling with CPSP. Summary: Modern approaches in managing CPSP require an interdisciplinary and patient-centric approach. Customizing treatment plans to address the specific etiology and symptoms of CPSP is crucial. Pharmacotherapy remains fundamental, encompassing medications such as anticonvulsants and antidepressants tailored to manage neuropathic pain. Integrating non-pharmacological interventions is crucial for providing comprehensive care. Additionally, investigating innovative technologies and personalized medicine presents promising opportunities to enhance treatment results and elevate the quality of life for those suffering from CPSP. Ultimately, an integrated approach that acknowledges the multifaceted nature of CPSP is essential for effective management and patient well-being.

## 1. Introduction

Central post-stroke pain (CPSP) is a focal, lesion-related pain that occurs continuously or intermittently in a part of the paralyzed body following hemorrhagic or ischemic stroke [1,2]. Usually, CPSP has a latency period, often developing within a few months following a stroke [3,4]. The occurrence of CPSP is closely linked to the location of the brain lesion, with lesions in the thalamus and brainstem being more likely to result in central neuropathic pain compared to other areas [5]. The clinical features of CPSP resemble those of other central and peripheral neuropathic pain conditions, lacking distinctive features or consistent signs in terms of onset, presentation, and intensity [6,7]. CPSP typically manifests as chronic pain described as burning, aching, or stabbing, often with a mix of sensory hyposensitivity and hypersensitivity in the painful area. The pain can be spontaneous or evoked, with intermittent dysesthesia occurring in up to 85% of patients [8,9]. Over 90% of patients exhibit abnormalities in thermal sensations, particularly cold, while sensory deficits in other modalities, such as touch and vibration, are less common [8,9,10,11].

The prevalence of CPSP in stroke patients ranges from 1% to 12% and can be as high as 55% [4,8]. However, the exact prevalence is uncertain due to variable clinical presentation, concurrent pain types, and the lack of clear diagnostic criteria. Namely, differentiating CPSP from other post-stroke pains, such as shoulder pain, painful spasticity, chronic headaches, and musculoskeletal conditions, is challenging [8,12,13,14,15,16]. Thus, diagnosing CPSP requires a comprehensive approach, including a detailed patient history, clinical and sensory examinations, imaging of lesions, and other clinical assessments. A definitive diagnosis is made only after ruling out other potential causes of nociceptive, psychogenic, or peripheral neuropathic pain [8,17,18].

Currently, there is minimal evidence connecting pain mechanisms, the site and nature of lesions, clinical presentations, and treatment outcomes [8,18]. Due to the lack of understanding, developing highly effective treatments for CPSP remains challenging. As such, current treatment options for CPSP are multifaceted, aiming to alleviate pain and improve functionality through a combination of pharmacological and non-pharmacological techniques, also inspired by evidence from similar clinical conditions [19,20,21]. Pharmacological treatment options include anticonvulsants like gabapentin and pregabalin, antidepressants such as amitriptyline and duloxetine, and/or other medications such as opioids [8,22,23]. Non-pharmacological strategies include physical therapy, transcutaneous electrical nerve stimulation (TENS), and cognitive-behavioral therapy (CBT), along with surgical methods such as deep brain stimulation (DBS) and invasive motor cortex stimulation (MCS) [8]. Furthermore, emerging treatments, including neuromodulation techniques and novel pharmacological agents, are also being explored to improve pain management and overall well-being for those affected by CPSP. Despite these advancements, CPSP remains a challenging condition to treat, necessitating ongoing research and innovation in therapeutic approaches. This narrative review examines the current methods for treating CPSP, highlighting both pharmacological and non-pharmacological approaches, with a focus on the mechanisms of action and effectiveness in clinical settings of these treatment options.

## 2. Mechanisms Underlying CPSP

As previously mentioned, CPSP is a chronic pain condition following a stroke, involving complex mechanisms that are not entirely clear. Several theories have been suggested to explain the underlying mechanisms of CPSP, highlighting the intricate nature of pain processing in the central nervous system [16]. One of the earliest explanations is the disinhibition theory [24,25]. It posits that damage to the lateral thalamus results in the loss of cortical control over thalamic activity, leading to thalamic hyperactivity and an exaggerated response to sensory stimuli. This hyperactivity manifests as CPSP due to the removal of inhibitory signals from the cortex, which allows for uncontrolled excitatory output from the thalamus [18,25]. Other research suggests that injury to the spinothalamic pathway is a key factor in the development of CPSP [18]. Boivie et al. in 1989 demonstrated that lesions at any level of this pathway could trigger CPSP, indicating its importance in pain transmission [9]. It was later shown that damage to the spinothalamocortical pathway is a significant predictor of CPSP development, independent of the lesion site [26]. Gritsch et al. found that central pain is due to hyperexcitability in the lateral thalamus, linked to calcium-voltage-dependent channels and changes in the GABAergic system [27]. Other studies showed increased connections between the thalamus and amygdala, and found abnormal activity between the medial thalamus and cingulate cortex, mediated by brain-derived neurotrophic factor (BDNF), involving imbalances in GABAergic and glutamatergic systems. This imbalance contributes to thalamocortical dysrhythmia, a characteristic feature of CPSP [18]. Another prominent hypothesis involves dysfunctional changes in brain plasticity following a stroke. It proposes that maladaptive neuroplasticity leads to pathological phenomena, such as spontaneous pain and hypersensitivity, driven by cortical and thalamic hyperexcitability [18,28].

Neuroimaging studies have identified structural and functional changes in the brain associated with CPSP. Functional MRI (fMRI) reveals increased activity in pain-related brain regions like the thalamus and somatosensory cortex during CPSP episodes. Whether or not such imaging, e.g., with radiomic texture analysis mapping, can predict clinical courses of CPSP, as shown for other applications, remains to be evaluated [29]. Diffusion tensor imaging (DTI) shows disrupted connectivity in white matter tracts linking these regions, potentially contributing to pain perception. Additionally, positron emission tomography (PET) scans indicate alterations in neurotransmitter systems, including increased opioid receptor binding in pain-processing areas. These findings highlight the complex neurobiological mechanisms underlying CPSP, emphasizing the need for further research to develop targeted treatments [8,30,31].

### 2.1. Pharmacologic Treatments for Central Post-Stroke Pain

Pharmacologic treatments for CPSP focus on mitigating neuropathic pain following a stroke. This presents diverse challenges, prompting the use of various medications targeting different pain mechanisms. Anticonvulsants, such as pregabalin and gabapentin, are commonly prescribed due to their ability to modulate neuronal excitability and reduce pain signals [8,32,33].

Antidepressants like amitriptyline and duloxetine are also effective, primarily by enhancing neurotransmitter levels involved in pain modulation [6,8]. Additionally, analgesics and other adjunctive therapies are utilized to manage CPSP symptoms comprehensively [8]. Despite these treatment options, the number of randomized controlled trials specifically targeting CPSP therapy is limited. The variability in treatment response underscores the need for interdisciplinary, individualized approaches to optimize CPSP management strategies [5].

#### 2.1.1. Antidepressants

Treatment of CPSP involves antidepressants like tricyclic antidepressants (TCAs) and selective serotonin-norepinephrine reuptake inhibitors (SNRIs). These medications alleviate pain by blocking neurotransmitter reuptake, enhancing descending inhibitory pain pathways in the central nervous system [34]. Amitriptyline, a TCA, is widely supported as the first-line treatment for CPSP, typically administered at a dose of 75 mg per day [5,8]. It has demonstrated significant efficacy, with a recent study indicating that 65% of CPSP patients experienced substantial pain relief [35]. While TCAs are commonly used in CPSP, they are not without side effects. Common issues include dry mouth, dizziness, and sedation, along with less frequent but more serious cardiovascular concerns such as orthostatic hypotension. In cases where amitriptyline is not well tolerated, alternatives with adrenergic properties, such as nortriptyline, desipramine, imipramine, and venlafaxine, are sometimes prescribed [5,36]. These TCAs are recognized for their ability to manage neuropathic pain; however, their effectiveness in individuals with CPSP remains unknown [5]. Alternatively, duloxetine, an SNRI, has recently demonstrated a 30% improvement in pain scores and enhanced quality of life for stroke survivors [37]. However, a recent study found that citalopram, another serotonergic antidepressant, had no effect in treating patients with CPSP [22]. Therefore, the treatment of CPSP with antidepressants requires a nuanced approach, taking into account the individual patient’s response to medication and the balance of efficacy and side effects.

#### 2.1.2. Anticonvulsants

Antiepileptic drugs (AEDs) are widely regarded as the drug of choice for the treatment of neuropathic pain syndromes and are increasingly used in the management of CPSP [5]. AEDs such as gabapentin and pregabalin are particularly notable for their efficacy in reducing pain severity and enhancing the overall well-being of CPSP patients [1,38,39,40]. These medications function by modulating calcium channel activity, thereby decreasing neuronal excitability and pain transmission [41]. Clinical studies have demonstrated significant pain relief in CPSP patients treated with gabapentin. Namely, a randomized controlled trial reported that 71% of patients experienced at least a 50% reduction in pain scores compared to 33% in the placebo group [42]. Similarly, pregabalin has shown promise, with a study indicating that 63% of patients experienced substantial pain relief and 47% reported improved sleep quality [43]. Despite these benefits, gabapentin and pregabalin also display mild side effects, including dizziness and somnolence [44]. However, it boasts an improved safety profile, making it a preferred alternative or combination therapy for managing neuropathic pain in patients who cannot tolerate higher doses of TCAs [35].

Alternatively, lamotrigine has shown promise in the treatment of CPSP, although its effectiveness varies [39]. In a double-blind, placebo-controlled crossover study, lamotrigine at 200 mg per day was administered to 30 patients with CPSP, resulting in a significant reduction in pain intensity in 42% of the participants compared to placebo [45]. Another study reported that 50% experienced at least a 30% reduction in pain scores with lamotrigine treatment [46]. Despite these positive outcomes, side effects such as dizziness and rash limit its use in some patients [5,39]. Overall, while lamotrigine can be beneficial for some individuals with CPSP, its variable efficacy and side effect profile necessitate careful patient selection and monitoring [39,46]. Furthermore, phenytoin has demonstrated some success, though research is limited [47]. Although AEDs are not universally effective for all CPSP patients, they represent a critical component of the multimodal approach to managing this debilitating condition.

#### 2.1.3. Opioids

While opioids such as morphine can alleviate neuropathic pain, they are not considered first-line medications [48,49]. A study involving a mixed neuropathic pain population, including 10 patients with CPSP, showed that oral opioids significantly reduced pain, with an average decrease of 23%. However, there was a high withdrawal rate among CPSP patients, who also reported experiencing less benefit from the treatment [50]. Another randomized placebo-controlled study on the effects of intravenous morphine in central pain showed no significant difference between morphine and placebo groups in pain relief outcomes [51]. It has been suggested that reduced opioid receptor binding in pain processing circuits may contribute to the inconsistent effects of morphine in treating CPSP [52]. Additionally, two studies examined the effect of the opioid antagonist naloxone on CPSP and found that intravenous naloxone did not prove effective in reducing CPSP symptoms [53,54]. As such, although opioids can alleviate neuropathic pain, their inconsistent effectiveness and high withdrawal rates in CPSP patients suggest they are still not optimal for treating CPSP. In addition, significant side effects must be considered [55].

#### 2.1.4. Alternative Pharmacotherapy

Several other drugs have shown evidence of efficacy in managing CPSP. Less commonly used options for treating central post-stroke pain include pamidronate, steroids, lidocaine, and ketamine [56,57]. A recent network meta-analysis found significant pain reduction with medications like pamidronate, prednisone, and levetiracetam, even ranking them as the top three most effective treatments [38]. Ketamine’s effectiveness in CPSP remains uncertain due to limited studies [58,59,60]. Lidocaine has provided only modest pain relief [38]. While not extensively researched for CPSP, cannabis-derived products and topical treatments have demonstrated efficacy in alleviating neuropathic pain [61]. However, due to a lack of longitudinal data, the long-term efficacy of these drugs still remains unknown [8].

### 2.2. Non-Pharmacologic Treatments

At present, there are two invasive methods for treating CPSP: deep brain stimulation (DBS) and invasive motor cortex stimulation (MCS). These neuromodulation techniques are commonly employed and are deemed safer than lesioning surgeries due to their reversibility [62]. Additionally, in pharmacoresistant patients, non-invasive treatments such as repetitive transcranial magnetic stimulation, transcutaneous electrical nerve stimulation (TENS), and cognitive behavioral therapy (CBT) have shown beneficial effects [8,63,64,65,66,67].

#### 2.2.1. Deep Brain Stimulation

**Figure 1:**
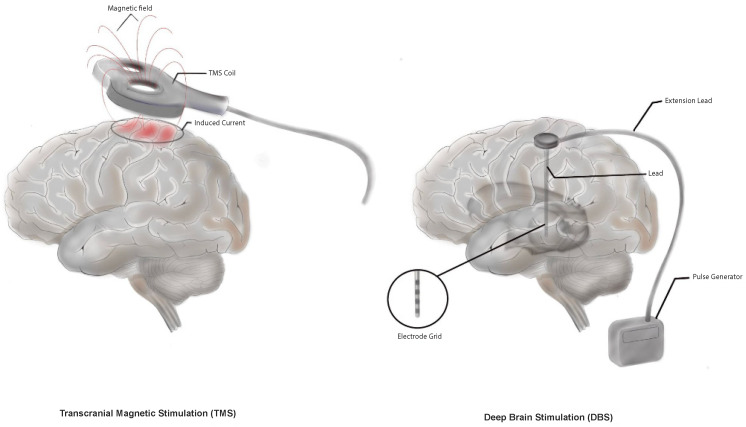
Depiction of Transcranial Magnetic Stimulation (TMS) and Deep Brain Stimulation (DBS) as treatments for central post-stroke pain (CPSP). TMS delivers non-invasive magnetic pulses to the brain’s cortical regions involved in pain control, while DBS involves surgically implanted electrodes that target deep brain structures to alleviate chronic pain. Both approaches aim to regulate abnormal neural activity contributing to CPSP symptoms.

DBS has emerged as a promising treatment for CPSP [68]. DBS involves the surgical implantation of electrodes into specific brain regions, typically targeting the thalamus or periaqueductal gray area, which are critical in pain processing (Figure 1) [69]. The electrodes deliver controlled electrical impulses that modulate neural activity, supposedly reducing pain perception and alleviating pain in CPSP patients [62,70,71]. The benefits of DBS include its reversibility and non-destructive nature and the ability to fine-tune treatment through adjustments of the stimulator settings post-implantation [63]. Reported efficacy rates range from 25% to 67% [71,72].

However, large multicenter trials aimed at evaluating the effectiveness of DBS for chronic pain relief fell short of the efficacy criteria, which required that at least half of the patients report a 50% reduction in pain one year post-surgery [73]. Consequently, the pursuit of FDA approval was discontinued, and DBS for pain management remains categorized as “off-label” [74].

In a prospective case series involving 18 patients with neuropathic pain, including 27.7% with CPSP, significant improvements were noted after DBS of the periaqueductal gray area and sensory thalamus [75]. Patients experienced an average reduction in subjective pain intensity of 44.7% [75]. Besides the thalamus and periaqueductal gray area, other target areas for DBS in CPSP patients have been described. For instance, a 2017 randomized controlled trial targeting the ventral striatum/anterior limb of the internal capsule in 10 participants indicated that half of them experienced at least a 50% reduction in pain intensity [76]. Additionally, patients reported improvements in their quality of life and functional abilities [76]. A recent study found that DBS targeting the central lateral thalamus, along with the ventral posterior complex, is a promising alternative when ventral posterior complex stimulation alone does not reduce pain satisfactorily [68].

While there is a possibility of technical implant failure or infections due to manipulation, the procedure is generally safe and allows patients to achieve significant pain relief, making DBS a viable option for pharmacoresistant patients [77].

#### 2.2.2. Motor Cortex Stimulation

In MCS, electrodes are implanted on a target area of the primary motor cortex, typically on the somatotopic site of the corresponding pain [76]. Once implanted, the electrodes deliver continuous electrical pulses to the motor cortex, modulating neuronal activity and thereby disrupting or altering the pain signals being processed in the brain [78]. The mechanisms underlying the effects of invasive MCS’s effects are not fully understood, but successful stimulation has been linked to changes in cerebral blood flow, affecting regions such as the thalamus [79,80]. A previous systematic review including data from 193 patients with CPSP treated with MCS suggested an initial positive response rate of 64%, which decreased to 55% at follow-up [81]. Two recent reviews estimated a one-year success rate of approximately 45–50% in CPSP patients [82,83].

A recent study including 16 participants demonstrated a mean Visual Analog Scale (VAS) score before surgery that was significantly higher than that of the last follow-up [84]. As for MCS, severe complications are infrequent and primarily related to infections, seizures and hardware malfunctions [82]. The effectiveness of MCS seems to be less favorable for post-stroke pain than for pain resulting from spinal cord injury or peripheral neuropathy [83,85]. As such, MCS shows promise in alleviating pain through modulation of neuronal activity, despite an incomplete understanding of its mechanisms and varying success rates across different types of pain disorders, necessitating further research to evaluate its long-term efficacy and safety.

#### 2.2.3. Transcranial Magnetic Stimulation

Repetitive TMS is a non-invasive method that activates targeted areas of the cerebral cortex by delivering magnetic pulses via coils positioned on the cranium (Figure 1) [86]. Recent trials investigating the effects of repetitive TMS on CPSP have showed mixed results, with some evidence indicating potential analgesic benefits, particularly for patients unresponsive to conventional therapies [87]. However, due to the novelty of this method, there is a significant lack of standardized protocols, particularly regarding the targeted cerebral area [82]. Patients with CPSP persisting beyond six months, inadequately controlled by two or more medications, and with VAS scores > 5 are typically candidates for TMS treatment [87]. Notably, it remains unclear which areas of the brain should be targeted, as most treatments focus on the primary motor cortex (M1), while some have explored the dorsolateral prefrontal cortex (DLPFC) and secondary somatosensory cortex (S2) [87]. Recent studies indicate that high-frequency repetitive TMS applied to the M1 can significantly reduce pain intensity in CPSP patients [87]. Additionally, a recent randomized controlled trial demonstrated that repetitive TMS not only improved pain outcomes but also enhanced patients’ quality of life and functional status over a 12-week period [88]. However, A recent review and meta-analysis suggest that the available evidence supporting the effectiveness of rTMS in reducing CPSP is of low quality. [89] The stimulation of the DLPFC for CPSP has shown some promise, though its efficacy is less well established compared to M1 stimulation [90]. While some studies have reported negative results, a study found that repetitive TMS targeting the DLPFC resulted in a 20% reduction in pain intensity on the VAS [91,92]. Notably, TMS targeting the S2 area has resulted in a significant long-term pain intensity reduction of approximately 15% [93]. Despite these encouraging findings, further investigation is needed to determine the long-term efficacy and to establish standardized guidelines for TMS treatment in CPSP, particularly for longer periods of time [87].

#### 2.2.4. Transcutaneous Electrical Nerve Stimulation

Transcutaneous electrical nerve stimulation (TENS) is a non-invasive method that stimulates nerves by delivering electrical impulses through the skin, thereby modulating pain signals and enhancing endorphin release [94]. TENS is an affordable, non-invasive, self-applied technique that is utilized as a supplement to medication, with some studies indicating its potential in managing CPSP [94,95]. Nevertheless, there are only a limited number of clinical trials examining TENS for central neuropathic pain. Most of these studies are either non-randomized or lack control groups, leading to varied outcomes [93]. A study on electrical stimulation for post-stroke shoulder pain discovered that high-intensity TENS, administered at three times the sensory threshold, was more effective than TENS at the sensory threshold and placebo TENS in alleviating hemiplegic shoulder pain and enhancing the passive range of motion for flexion [95,96]. Moreover, a meta-analysis of eight studies revealed that functional electrical stimulation and TENS significantly enhanced gait speed in post-stroke patients. However, the studies differed in terms of the stimulation devices used, electrode placement, and dosage [97,98]. TENS caused a temporary increase in pain in one-third of the patients [98]. While TENS has shown potential in treating CPSP, it is important to state that TMS and TDCS have shown better results [94]. Further investigation is necessary to establish the effectiveness of TENS.

#### 2.2.5. Cognitive Behavioral Therapy

Research into psychological interventions, particularly cognitive behavioral therapy (CBT), has shown significant promise in addressing the complex nature of chronic pain conditions, including chronic post-stroke pain (CPSP) [99]. CBT aims to modify dysfunctional thoughts and behaviors that exacerbate pain perception and persistence [100]. By teaching patients adaptive coping strategies and stress management techniques, CBT enhances their ability to manage pain effectively and improve their overall quality of life [101]. Studies highlight that CBT not only reduces pain severity but also helps in reducing disability associated with CPSP by empowering patients to regain control over their lives and activities despite ongoing pain [100]. This is crucial for individuals with CPSP who often experience limitations in daily activities due to pain.

Additionally, CBT addresses common comorbidities such as anxiety and depression that frequently accompany chronic pain conditions, including those following a stroke [100,102]. By targeting these psychological symptoms, CBT contributes to a comprehensive treatment approach that improves overall patient well-being and mental health outcomes [103].

Furthermore, the holistic nature of CBT aligns with the biopsychosocial model of pain management, which recognizes the interplay between biological, psychological, and social factors in shaping pain experiences [104]. This approach not only treats symptoms but also addresses the underlying psychological factors contributing to pain chronicity, thereby offering long-term benefits for patients with CPSP [105].

In conclusion, CBT represents a valuable therapeutic tool in the multidisciplinary management of chronic post-stroke pain [106]. Its ability to enhance coping skills, reduce pain severity, and improve functional outcomes underscores its role in promoting a holistic approach to patient care and well-being.

#### 2.2.6. Virtual Reality

In recent years, virtual reality (VR) has gained traction as a tool in treating chronic pain forms such as post-stroke pain, offering an immersive and interactive approach to pain management. By engaging patients in virtual environments, VR can redirect their attention away from pain, creating a distraction that reduces their perception of discomfort [107]. Additionally, VR-assisted therapy often involves motor tasks that encourage movement and rehabilitation, contributing to joint mobility and function while simultaneously lessening pain [107,108].

To date, there is only limited amount of research involving the application of VR in post-stroke pain relief. A recent umbrella review on the use of VR against chronic pain including 21 systematic reviews reported positive results. Of them, ten studies reported benefits beyond pain improvement such as positive effects in anxiety and overall mental health as well as physical function [109]. Another study involving 20 stroke patients compared VR-assisted physical therapy to conventional physical therapy alone, revealing a reduction in pain as well as an improved joint range of motion in the lower extremities [110]. A study involving ten stroke patients with pain showed that all applied VR conditions had distinct effects on altering their pain threshold to both hot and cold stimuli [110]. One study suggested that VR could be beneficial in home rehabilitation by demonstrating a reduction in hemiplegic shoulder pain in a stroke patient [111].

Hence, while the research on the use of VR for CPSP relief is still limited, early findings are promising. VR not only offers pain reduction but also contributes to improvements in physical function, joint mobility, and even mental well-being [108]. Although VR is generally considered to be a safe and non-invasive procedure with few side effects, it is important to mention that the use of VR can induce side effects such as VR sickness, nausea and headache [112]. Nevertheless, VR stands as a valuable, non-invasive tool in the broader landscape of post-stroke rehabilitation, with the capacity to enhance recovery outcomes both physically and mentally [107,113]. Continued research is necessary to fully understand its long-term benefits and optimize its application in this field.

#### 2.2.7. Desensitization Therapy

Desensitization therapy, a technique aimed at reducing hypersensitivity to pain, has shown promise in treating CPSP [114]. This approach involves gradually exposing patients to stimuli that evoke discomfort in a controlled and systematic manner, helping to retrain the nervous system and decrease pain sensitivity over time [115].

By progressively increasing the tolerance to sensory inputs and promoting neural adaptation, desensitization therapy aims to alleviate the heightened pain response often experienced by stroke survivors. The therapy may include techniques such as graded exposure to various textures, temperatures, and pressures, combined with cognitive behavioral strategies to help patients manage and reduce their pain perception [114,115].

Although research on the effectiveness of desensitization therapy for CPSP is still limited, recent evidence suggests its potential benefits. For instance, a recent case report indicated that desensitization therapy resulted in a 40% improvement in pain sensitivity on the right side of the body compared to pre-test levels [116]. The study concluded that desensitization techniques effectively reduced pain and enhanced sensation in patients with thalamic pain syndrome [116]. Nonetheless, further research is necessary to fully validate these findings and refine desensitization techniques for broader use in CPSP treatment.

## 3. Outcome and Outlook

The pathophysiological mechanisms underlying CPSP, such as central sensitization and cortical reorganization, have been elucidated. Pharmacological treatments, including antidepressants and anticonvulsants, are commonly recommended as first-line therapies in managing CPSP. Antidepressants like amitriptyline and duloxetine work by modulating neurotransmitters to alleviate pain, while anticonvulsants such as gabapentin and pregabalin reduce pain by affecting neuronal excitability. Emerging pharmacological targets, such as cannabinoids or topical treatments, show promise, though their efficacy is still under investigation due to the limited number of robust studies. Non-pharmacological approaches such as TMS and physical therapy modalities demonstrate efficacy in alleviating CPSP symptoms. Invasive interventions like MCS and DBS show potential for refractory cases. While MCS and DBS offer promising results in pain reduction, research continues to explore novel therapies like repetitive TMS and TENS. Psychological interventions like CBT also play a crucial role in improving coping skills and overall quality of life for CPSP patients, addressing both pain severity and associated mental health challenges within a biopsychosocial framework. Despite these advancements, contradictions in current research highlight the need for further studies to refine treatment protocols. While antidepressants and anticonvulsants are recommended as effective initial therapies, the variability in patient response and the limitations of existing studies suggest that a more individualized, multimodal approach is necessary. Continued research is essential to enhance treatment efficacy and establish standardized protocols for the effective management of CPSP.

## 4. Conclusions

Recent advancements in treatment approaches for CPSP provide hope for improved outcomes in stroke survivors. Although antidepressants and anticonvulsants are generally recommended as effective initial treatments, the variability in patient responses and the limitations of current research indicate that a more personalized, multimodal approach is required. Personalized therapies based on individual patient characteristics and the exploration of novel targets hold promise for optimizing CPSP management. However, further research is needed to address existing challenges and refine treatment strategies. This narrative review serves as a roadmap for clinicians and researchers, guiding efforts to enhance the care and quality of life of individuals living with CPSP.

## Abbreviations

AEDs	Antiepileptic drugs
CBT	Cognitive behavioral therapy
CPSP	Central post-stroke pain
DBS	Deep brain stimulation
DLPFC	Dorsolateral prefrontal cortex
DTI	Diffusion tensor imaging
fMRI	Functional MRI
MCS	Motor cortex stimulation
M1	Primary motor cortex
PET	Positron emission tomography
SNRIs	Serotonin-norepinephrine reuptake inhibitors
S2	Secondary Somatosensory Cortex
TCA	Tricyclic Antidepressant
TENS	Transcutaneous electrical nerve stimulation
TMS	Transcranial magnetic stimulation
VAS	Visual Analog Scale
